# Supplementary material for: Harnessing protein language model for structure-based discovery of highly efficient and robust PET hydrolases
Source: Nat Commun. 2025 Jul 5;16:6211. doi: 10.1038/s41467-025-61599-z (PMC12228687; doi:10.1038/s41467-025-61599-z)
Supplement: Supplementary file 1 — Supplementary Information [file 41467_2025_61599_MOESM1_ESM.pdf]

Supplementary Information for:

Harnessing Protein Language Model for Structure-Based Discovery of Highly Efficient and Robust PET Hydrolases

Banghao Wu[1,2,4†], Bozitao Zhong[1,2†], Lirong Zheng[2,5*], Runye Huang[1,2,4], Shifeng Jiang[1], Mingchen Li[2,3], and Liang Hong[1,2,3,4*], Pan Tan[1,2,3*]

1. School of Life Sciences and Biotechnology, Shanghai Jiao Tong University, Shanghai 200240, China
2. Shanghai National Center for Applied Mathematics (SJTU Center) & Institute of Natural Sciences, Shanghai Jiao Tong University, Shanghai 200240, China
3. Shanghai Artificial Intelligence Laboratory, Shanghai 200232, China
4. Zhang Jiang Institute for Advanced Study, Shanghai Jiao Tong University, Shanghai 201203, China.
5. Department of Cell and Developmental Biology & Michigan Neuroscience Institute, University of Michigan Medical School, Ann Arbor, Michigan, 48104, USA

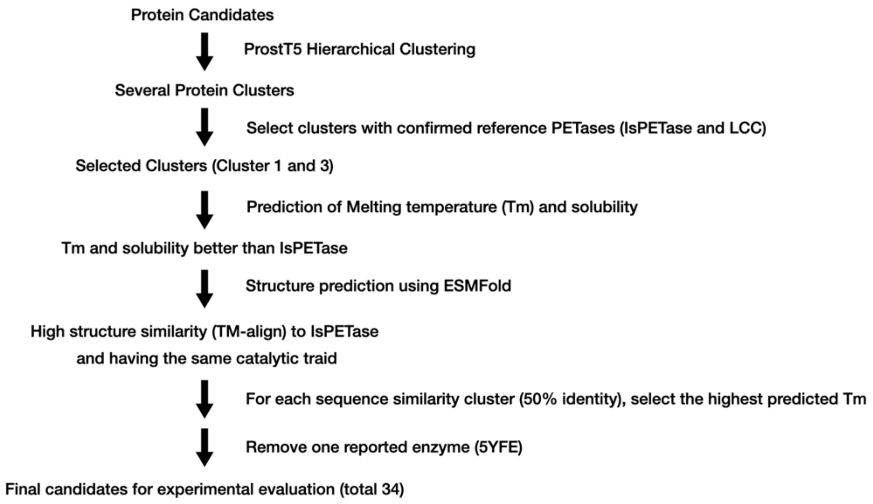

Supplementary Figure 1. Selection pipeline for discovered PET hydrolyses.

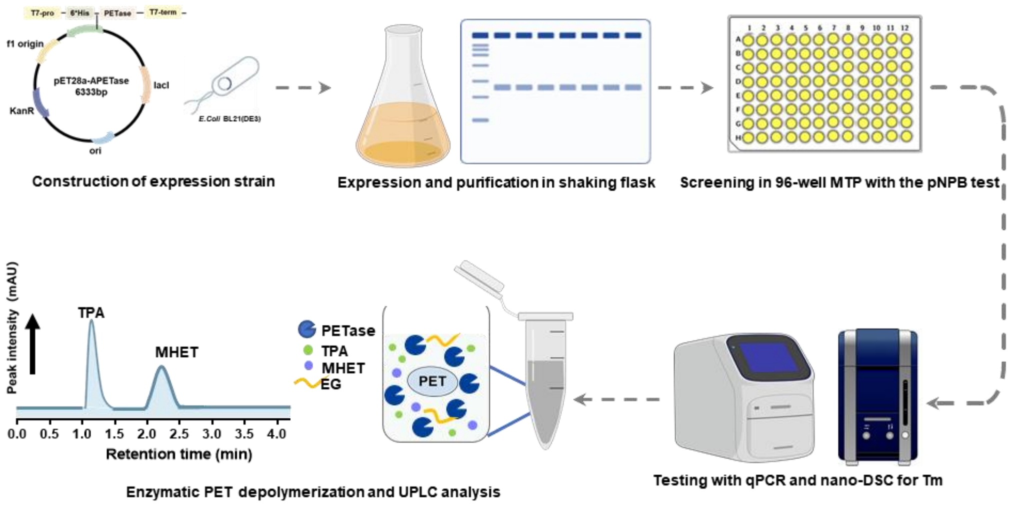

Supplementary Figure 2. Experimental validation workflow for the discovered PETases. Image provided by Servier Medical Art (https://smart.servier.com/), licensed under CC BY 4.0 (https://creativecommons.org/licenses/by/4.0/). The image was provided by or adapted from Bioicons (https://bioicons.com), licensed under under CC0 1.0 Universal Public Domain (https://creativecommons.org/public-domain/cc0/) and CC BY 4.0 (https://creativecommons.org/licenses/by/4.0/). Eppendorf_dilutionseries created by Andi-Wilson is licensed under CC-BY 4.0 Unported. Desktop_electron_microscope created by DBCLS is licensed under CC-BY 4.0. Electrophoresis-gel created by DBCLS is licensed under CC-BY 4.0 Unported.

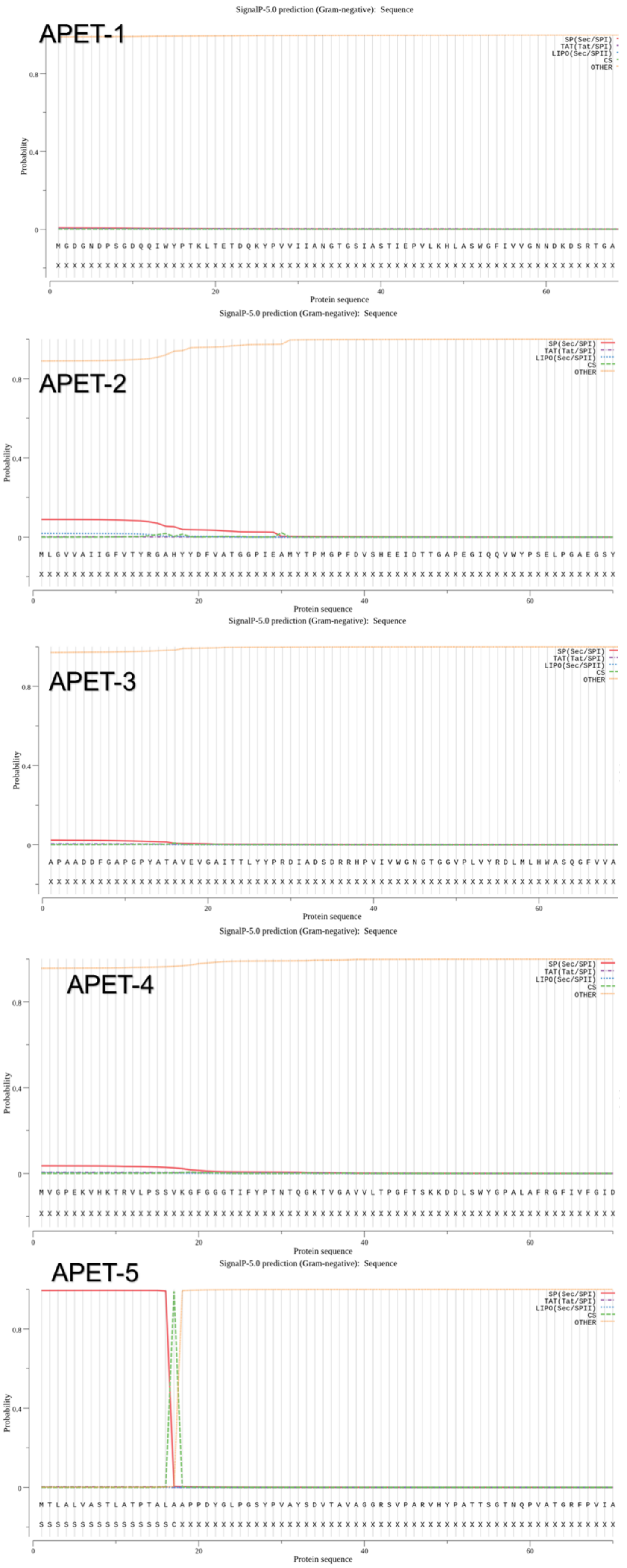
SignalP-5.0 prediction (Gram-negative): Sequence
APET-1
SP(Sec/SPI)
TAT(Tat/SPI)
LIPO(Sec/SPII)
CS
OTHER
Probability
M G D G N D P S G D Q Q I W Y P T K L T E T D Q K Y P V V I I A N G T G S I A S T I E P V L K H L A S W G F I V V G N N D K D S R T G A
Protein sequence
SignalP-5.0 prediction (Gram-negative): Sequence
APET-2
M L G V V A I I G F V T Y R G A H Y Y D F V A T G G P I E A M Y T P M G P F D V S H E E I D T T G A P E G I Q Q V W Y P S E L P G A E G S Y
Protein sequence
SignalP-5.0 prediction (Gram-negative): Sequence
APET-3
A P A A D D F G A P G P Y A T A V E V G A I T T L Y Y P R D I A D S D R R H P V I V W G N G T G G V P L V Y R D L M L H W A S Q G F V V A
Protein sequence
SignalP-5.0 prediction (Gram-negative): Sequence
APET-4
M V G P E K V H K T R V L P S S V K G F G G G T I F Y P T N T Q G K T V G A V V L T P G F T S K K D D L S W Y G P A L A F R G F I V F G I D
Protein sequence
APET-5
SignalP-5.0 prediction (Gram-negative): Sequence
M T L A L V A S T L A T P T A L A A P P D Y G L P G S Y P V A Y S D V T A V A G G R S V P A R V H Y P A T T S G T N Q P V A T G R F P V I A
S S S S S S S S S S S S S S S S C
Protein sequence

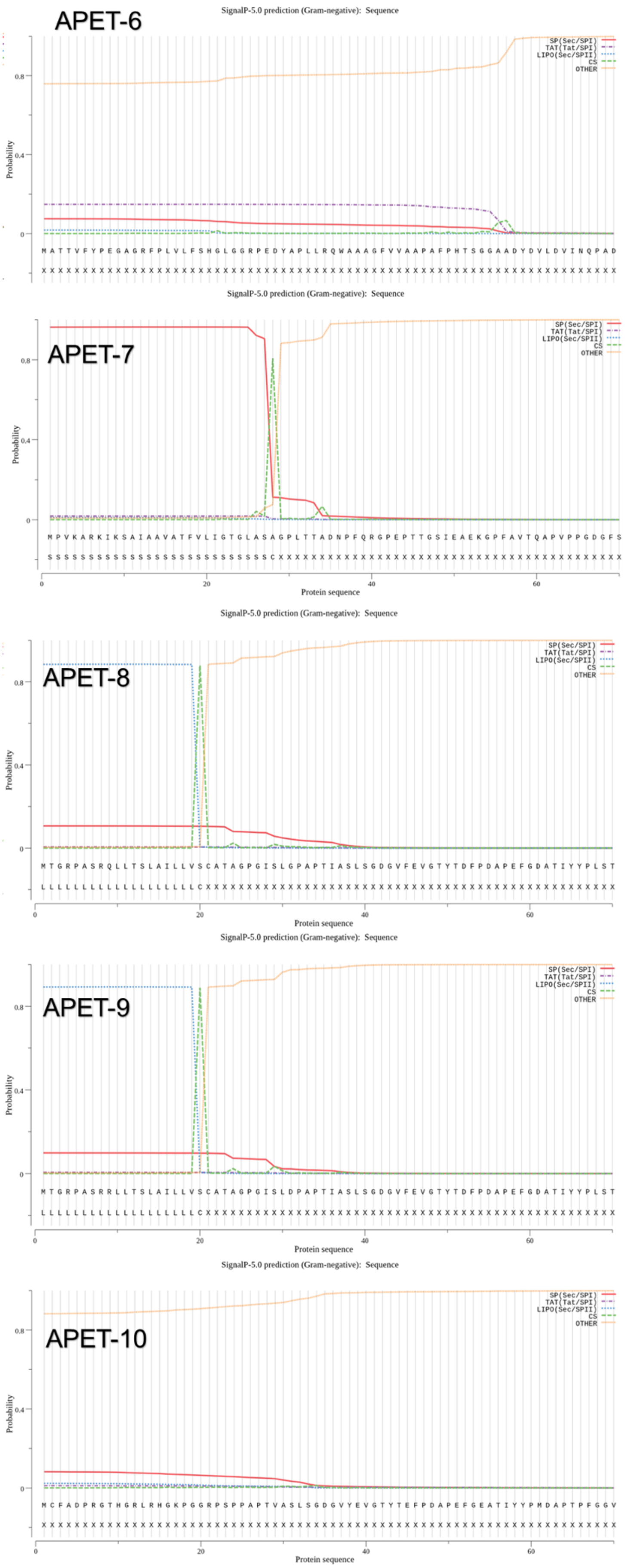
APET-6
SignalP-5.0 prediction (Gram-negative): Sequence
SP(Sec/SPI)
TAT(Tat/SPI)
LIPO(Sec/SPII)
CS
OTHER
Probability
0.8
0.4
0
MATTVFYPEGAGRFPLVLFSHGLGGRPEDYAPLLRQWAAAGFVVAAPAFPHTSGGAADYDVLDVINQPAD
XXXXXXXXXXXXXXXXXXXXXXXXXXXXXXXXXXXXXXXXXXXXXXXXXXXXXXXXXXXXXXXXXXXXXX
SignalP-5.0 prediction (Gram-negative): Sequence
APET-7
SP(Sec/SPI)
TAT(Tat/SPI)
LIPO(Sec/SPII)
CS
OTHER
Probability
0.8
0.4
0
MPVKARKIKSAIAAVATFVLIGTGLASAGPLTTADNPFQRGPEPTTGSIEAEKGPFAVTQAPVPPGDGFS
SSSSSSSSSSSSSSSSSSSSSSSSSSSCXXXXXXXXXXXXXXXXXXXXXXXXXXXXXXXXXXXXXXXXXX
0
20
40
60
Protein sequence
SignalP-5.0 prediction (Gram-negative): Sequence
APET-8
SP(Sec/SPI)
TAT(Tat/SPI)
LIPO(Sec/SPII)
CS
OTHER
Probability
0.8
0.4
0
MTGRPASRQLLTSLAILLVSCATAGPGISLGPAPTIASLSGDGVFEVGTYTDFPDAPEFGDATIYYPLST
LLLLLLLLLLLLLLLLLLLCXXXXXXXXXXXXXXXXXXXXXXXXXXXXXXXXXXXXXXXXXXXXXXXXXX
0
20
40
60
Protein sequence
SignalP-5.0 prediction (Gram-negative): Sequence
APET-9
SP(Sec/SPI)
TAT(Tat/SPI)
LIPO(Sec/SPII)
CS
OTHER
Probability
0.8
0.4
0
MTGRPASRRLLTSLAILLVSCATAGPGISLDPAPTIASLSGDGVFEVGTYTDFPDAPEFGDATIYYPLST
LLLLLLLLLLLLLLLLLLLCXXXXXXXXXXXXXXXXXXXXXXXXXXXXXXXXXXXXXXXXXXXXXXXXXX
0
20
40
60
Protein sequence
SignalP-5.0 prediction (Gram-negative): Sequence
APET-10
SP(Sec/SPI)
TAT(Tat/SPI)
LIPO(Sec/SPII)
CS
OTHER
Probability
0.8
0.4
0
MCFADPRGTHGRLRHGKPGGRPSPPAPTVASLSGDGVYEVGTYTEFPDAPEFGEATIYYPMDAPTPFGGV
XXXXXXXXXXXXXXXXXXXXXXXXXXXXXXXXXXXXXXXXXXXXXXXXXXXXXXXXXXXXXXXXXXXXXX
0
20
40
60
Protein sequence

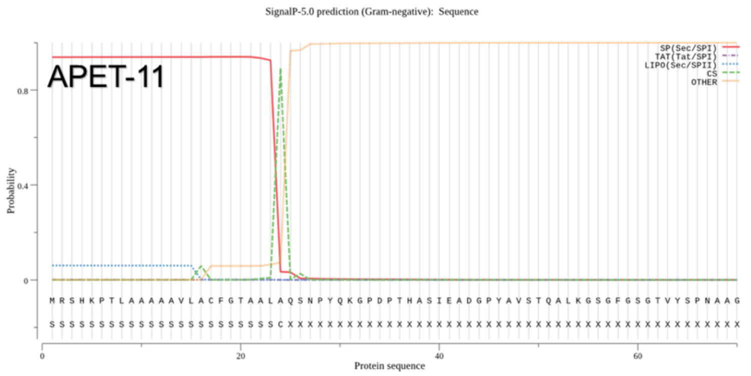
SignalP-5.0 prediction (Gram-negative): Sequence
APET-11
SP(Sec/SPI)
TAT(Tat/SPI)
LIPO(Sec/SPII)
CS
OTHER
Probability
0.8
0.4
0
M R S H K P T L A A A A A V L A C F G T A A L A Q S N P Y Q K G P D P T H A S I E A D G P Y A V S T Q A L K G S G F G S G T V Y S P N A A G
S S S S S S S S S S S S S S S S S S S S S S S C X X X X X X X X X X X X X X X X X X X X X X X X X X X X X X X X X X X X X X X X X X X X X X
0
20
40
60
Protein sequence

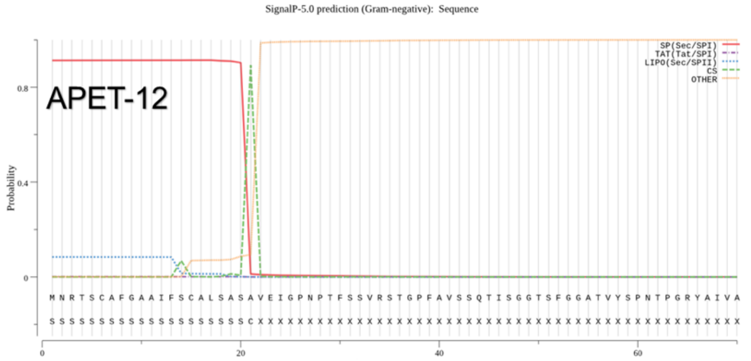
SignalP-5.0 prediction (Gram-negative): Sequence
APET-12
SP(Sec/SPI)
TAT(Tat/SPI)
LIPO(Sec/SPII)
CS
OTHER
Probability
0.8
0.4
0
M N R T S C A F G A A I F S C A L S A S A V E I G P N P T F S S V R S T G P F A V S S Q T I S G G T S F G G A T V Y S P N T P G R Y A I V A
S S S S S S S S S S S S S S S S S S S S C X X X X X X X X X X X X X X X X X X X X X X X X X X X X X X X X X X X X X X X X X X X X X X X X X
0
20
40
60

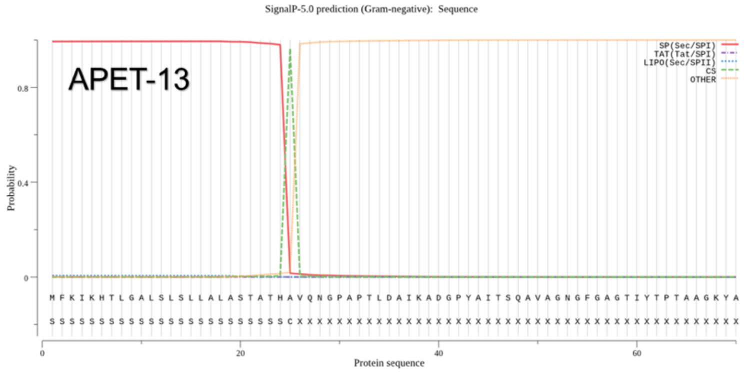
SignalP-5.0 prediction (Gram-negative): Sequence
APET-13
SP(Sec/SPI)
TAT(Tat/SPI)
LIPO(Sec/SPII)
CS
OTHER
Probability
0.8
0.4
0
M F K I K H T L G A L S L S L L A L A S T A T H A V Q N G P A P T L D A I K A D G P Y A I T S Q A V A G N G F G A G T I Y T P T A A G K Y A
S S S S S S S S S S S S S S S S S S S S S S S S C X X X X X X X X X X X X X X X X X X X X X X X X X X X X X X X X X X X X X X X X X X X X
0
20
40
60
Protein sequence

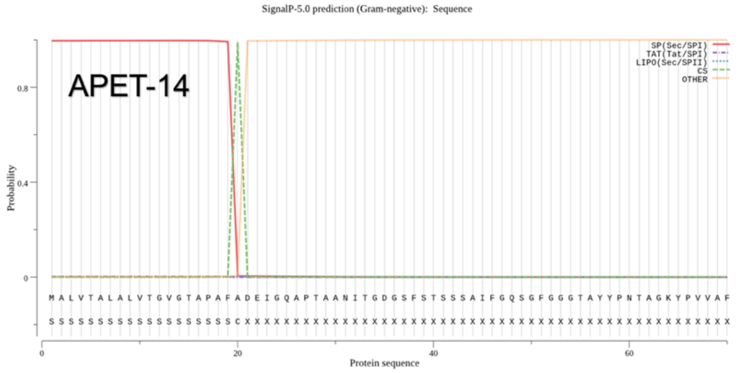
SignalP-5.0 prediction (Gram-negative): Sequence
APET-14
SP(Sec/SPI)
TAT(Tat/SPI)
LIPO(Sec/SPII)
CS
OTHER
Probability
0.8
0.4
0
M A L V T A L A L V T G V G T A P A F A D E I G Q A P T A A N I T G D G S F S T S S S A I F G Q S G F G G G T A Y Y P N T A G K Y P V V A F
S S S S S S S S S S S S S S S S S S S C X X X X X X X X X X X X X X X X X X X X X X X X X X X X X X X X X X X X X X X X X X X X X X X X X X
0
20
40
60
Protein sequence

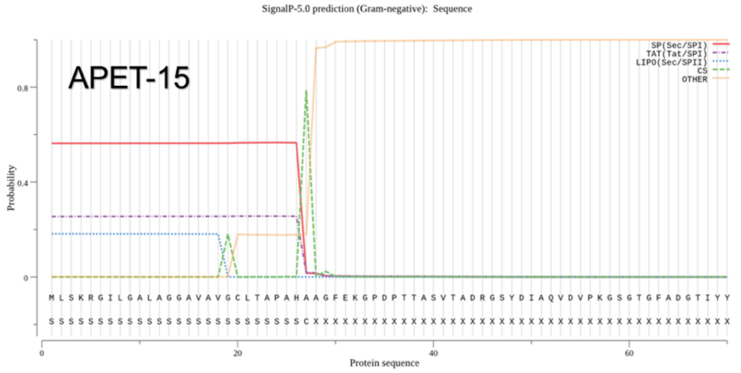
SignalP-5.0 prediction (Gram-negative): Sequence
APET-15
SP(Sec/SPI)
TAT(Tat/SPI)
LIPO(Sec/SPII)
CS
OTHER
Probability
0.8
0.4
0
M L S K R G I L G A L A G G A V A V G C L T A P A H A A G F E K G P D P T T A S V T A D R G S Y D I A Q V D V P K G S G T G F A D G T I Y Y
S S S S S S S S S S S S S S S S S S S S S S S S S S C X X X X X X X X X X X X X X X X X X X X X X X X X X X X X X X X X X X X X X X X X X X
0
20
40
60
Protein sequence

SignalP-5.0 prediction (Gram-negative): Sequence

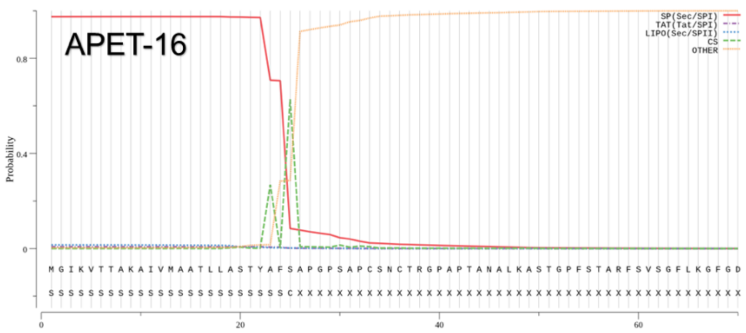

SignalP-5.0 prediction (Gram-negative): Sequence

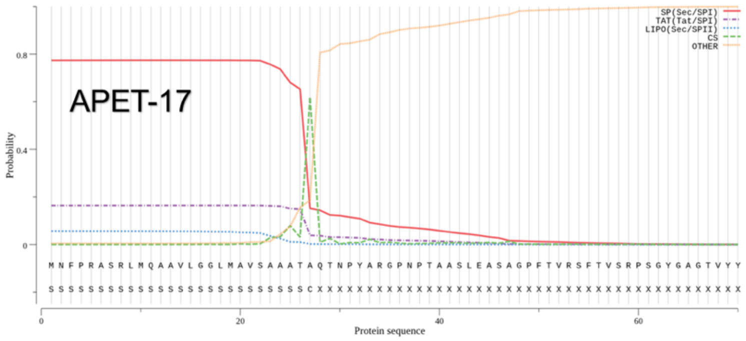

SignalP-5.0 prediction (Gram-negative): Sequence

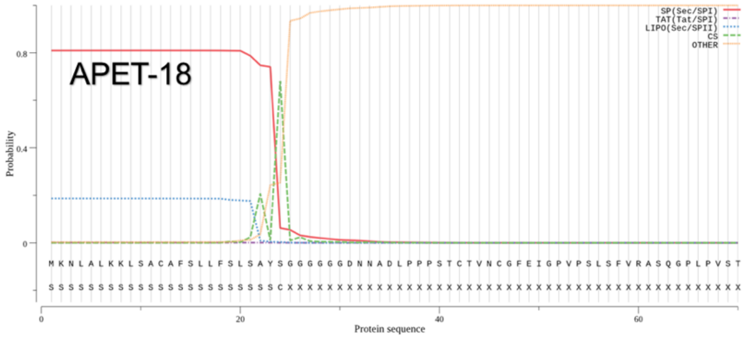

SignalP-5.0 prediction (Gram-negative): Sequence

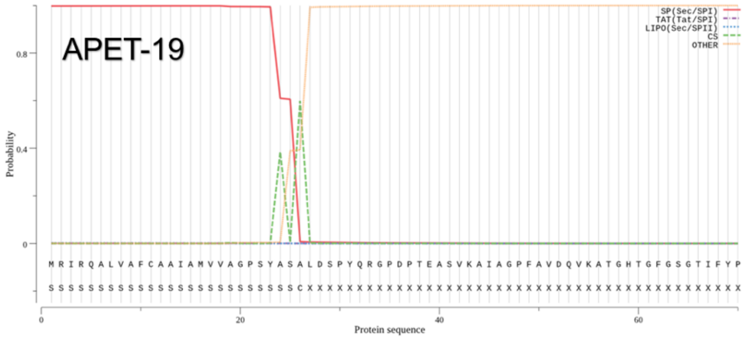

SignalP-5.0 prediction (Gram-negative): Sequence

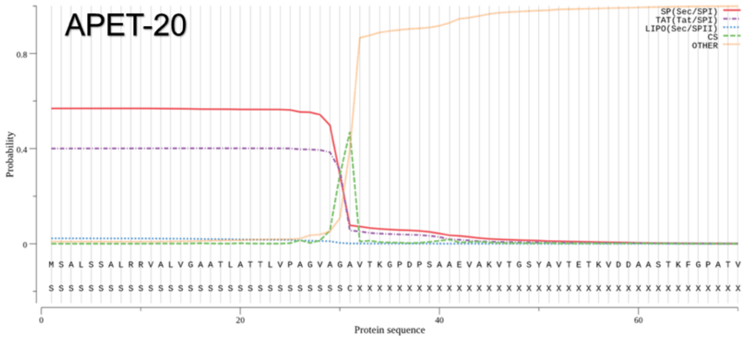

SignalP-5.0 prediction (Gram-negative): Sequence

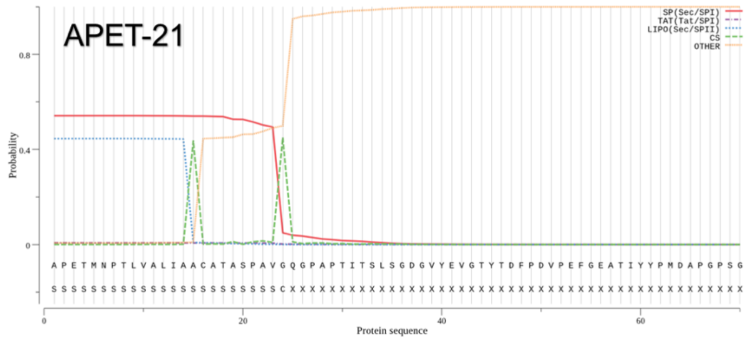

SignalP-5.0 prediction (Gram-negative): Sequence

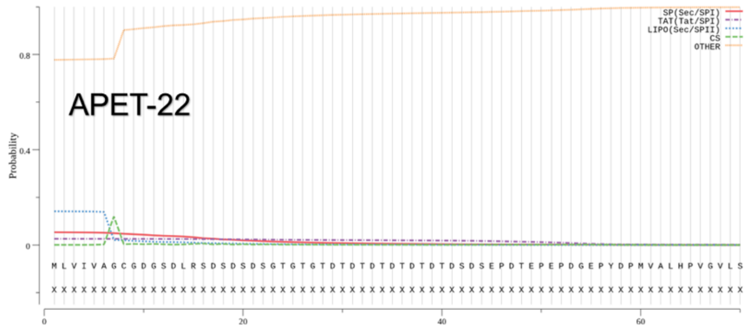

SignalP-5.0 prediction (Gram-negative): Sequence

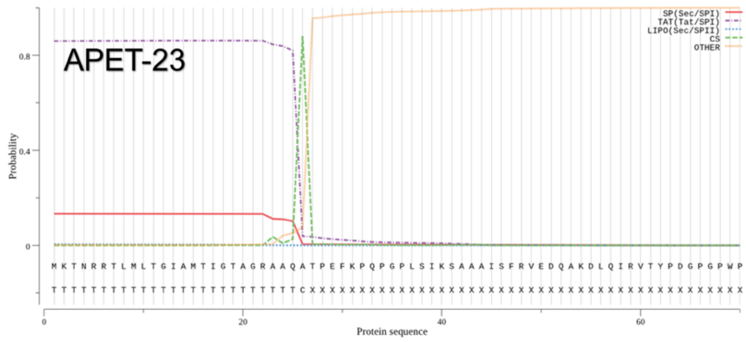

SignalP-5.0 prediction (Gram-negative): Sequence

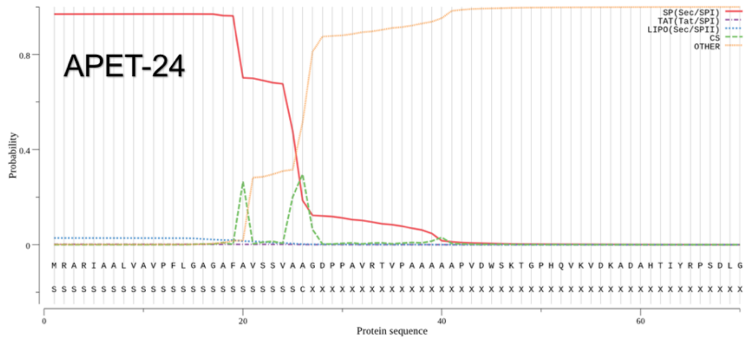

SignalP-5.0 prediction (Gram-negative): Sequence

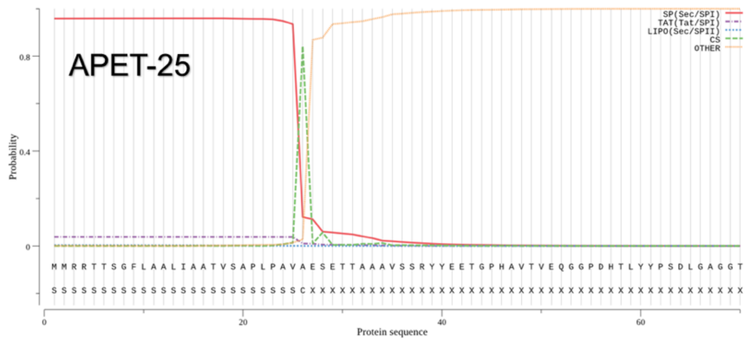

SignalP-5.0 prediction (Gram-negative): Sequence

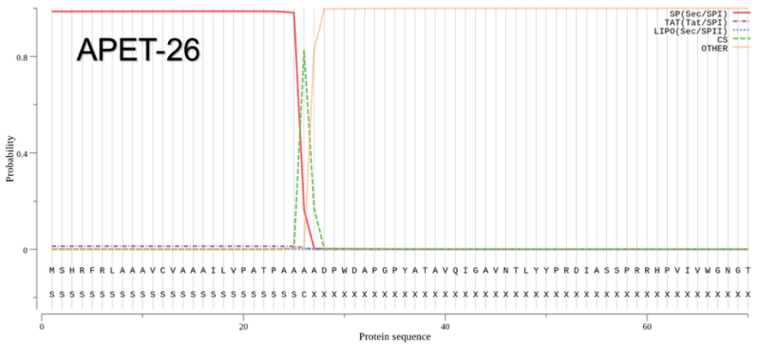

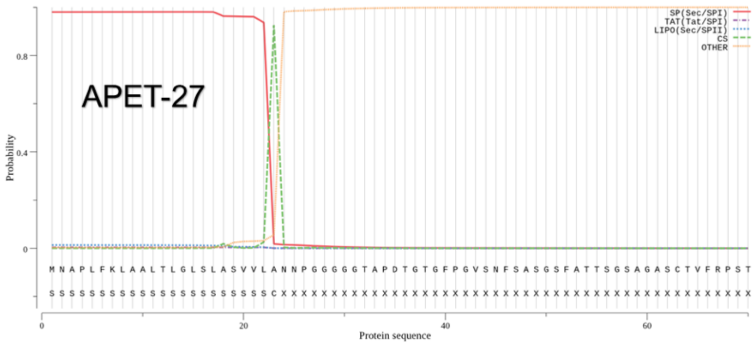

SignalP-5.0 prediction (Gram-negative): Sequence

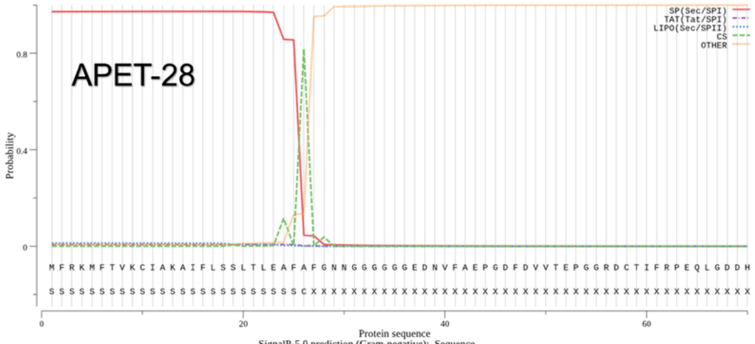

SignalP-5.0 prediction (Gram-negative): Sequence

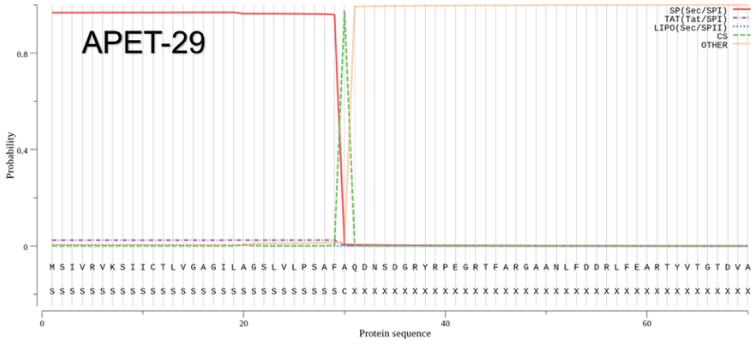

SignalP-5.0 prediction (Gram-negative): Sequence

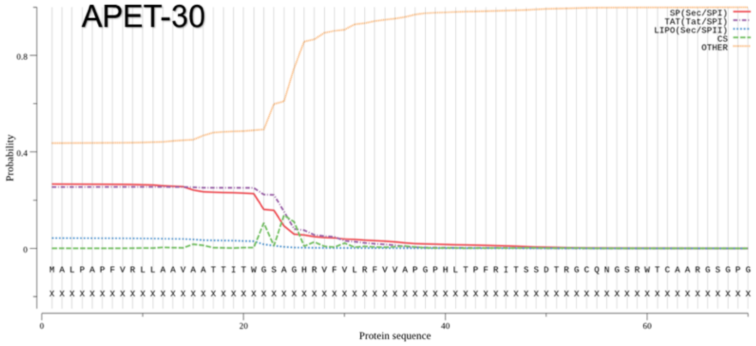

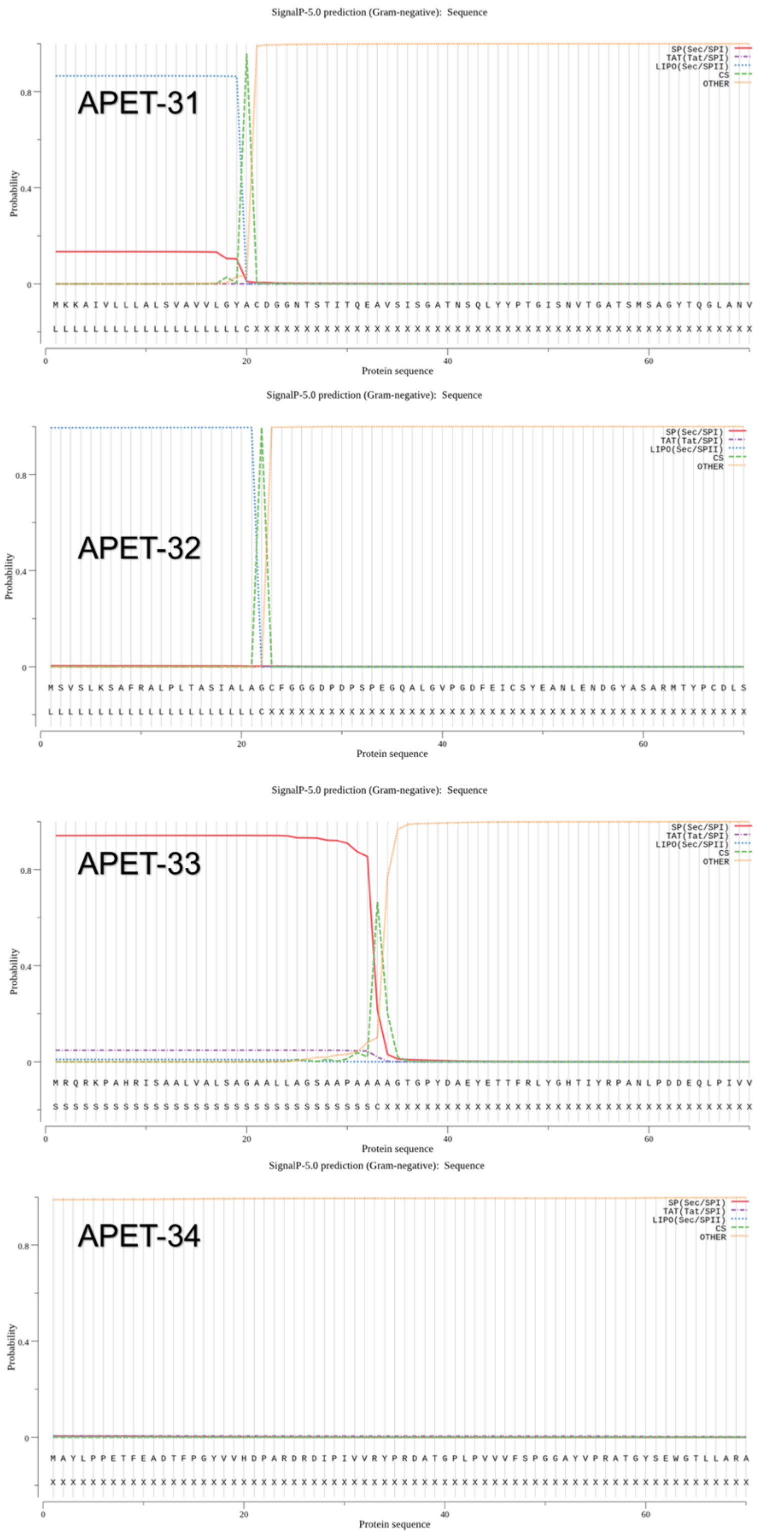

Supplementary Figure 3. The likelihood of presence of a signal peptide in APET-1-APET-34 sequences, as predicted by SignalP5.0[1]. APET-5, APET-7, APET11-21, APET-24-29 and APET-33 are predicted to contain Sec signal peptides cleavable by SPase (Sec/SPI). APET-8, APET-9, APET- 31 and APET- 32 are predicted to contain Sec (Lipoprotein) signal peptides cleavable by SPase (Sec/SPII). APET-23 is predicted to contain Tat signal peptides cleavable by SPase (Sec/SPI).

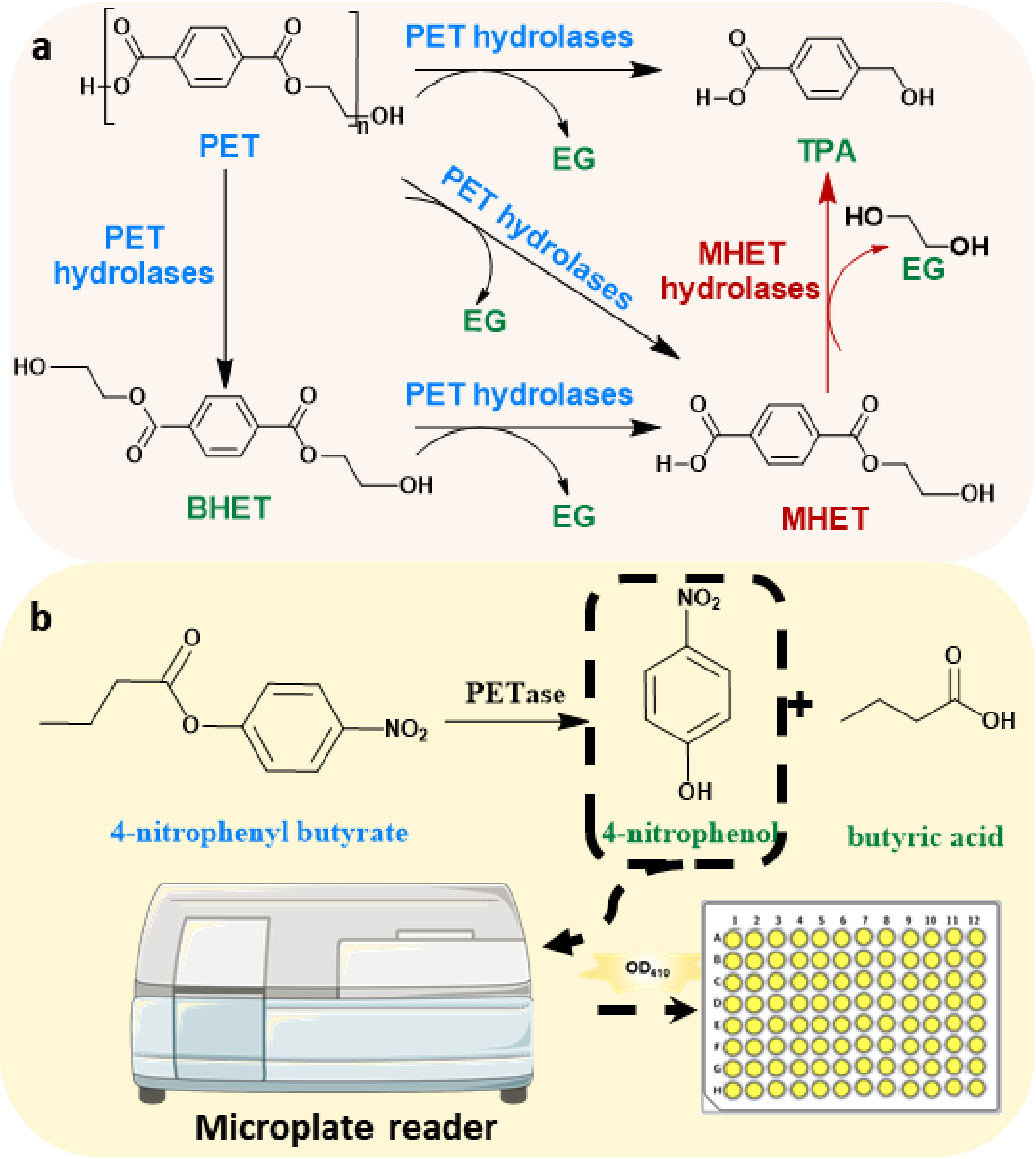

Supplementary Figure 4. The schematic for the initial screening of PET hydrolase candidates using the substrate p-nitrophenyl butyrate (*p*NPB) is illustrated as follows[2]. (a) The hydrolysis process of PET polymers; (b) shows that *p*NPB can be hydrolyzed by PETase, resulting in the formation of 4-nitrophenol and butyric acid. Notably, 4-nitrophenol exhibits a peak absorbance at 410 nm. By monitoring changes of OD_{410}, we can assess the ester bond hydrolysis activity of the candidate proteins, which serves as an indirect indication of their potential PET hydrolytic capabilities. Image provided by Servier Medical Art (https://smart.servier.com/), licensed under CC BY 4.0 (https://creativecommons.org/licenses/by/4.0/).

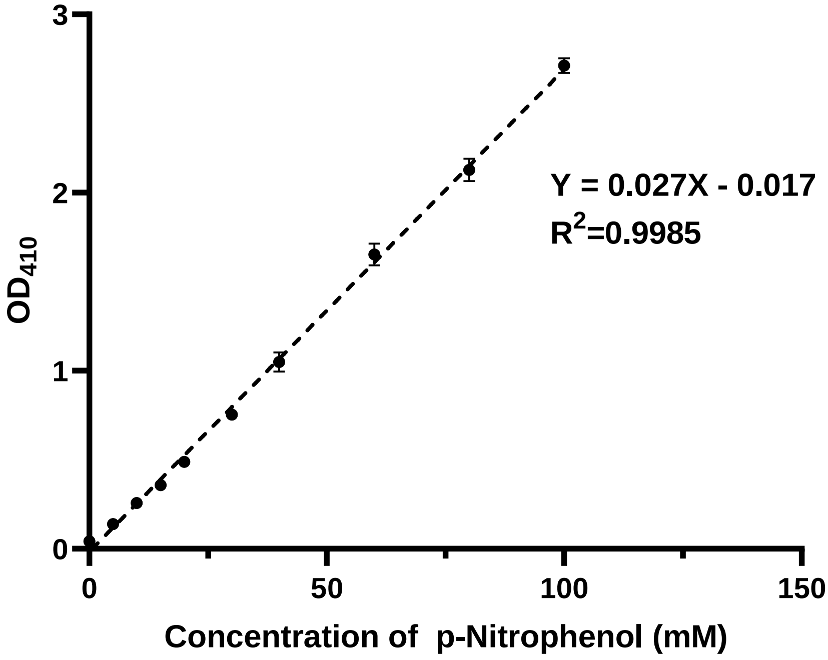

Supplementary Figure 5. The standard curve of p-nitrophenol, which is employed for the quantitative assessment of the ester bond hydrolytic capabilities of the candidate PETases[2]. By utilizing this methodology, we can effectively evaluate the hydrolytic activity of each candidate, providing insights into their potential efficiency in degrading PET substrates. Reactions were performed in triplicate; data are presented as mean values ± SD.

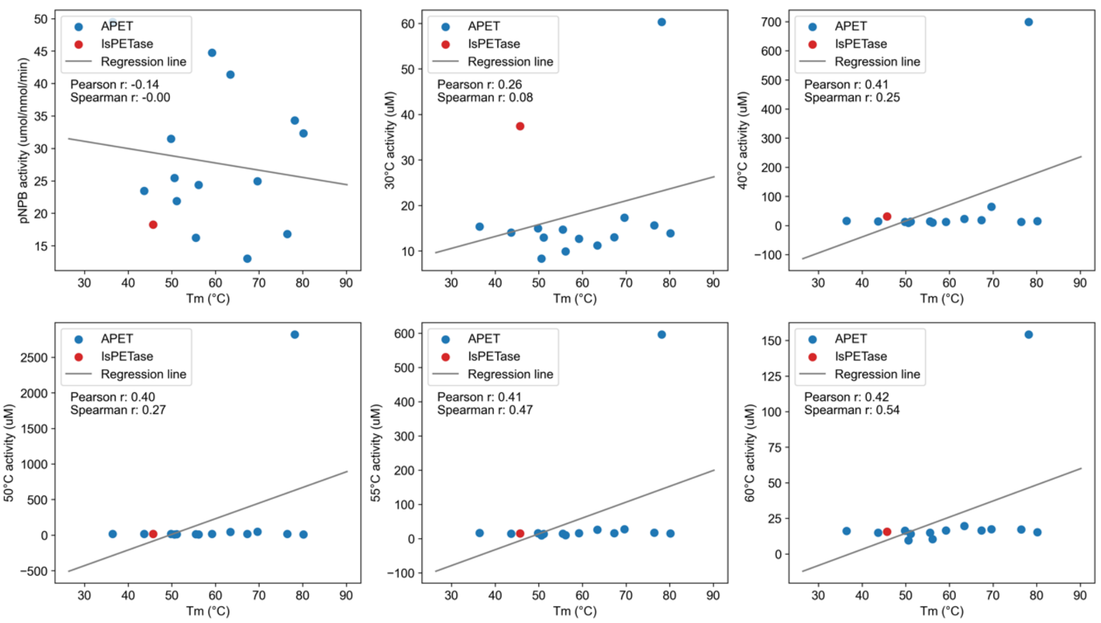

Supplementary Figure 6. Correlation between enzymatic activity and T_m for PETases shown in Fig. 2. IsPETase is highlighted in red, while the discovered APETase variants are shown in blue. A linear regression line (gray), along with Pearson (r) and Spearman (ρ) correlation coefficients, is included in the figure.

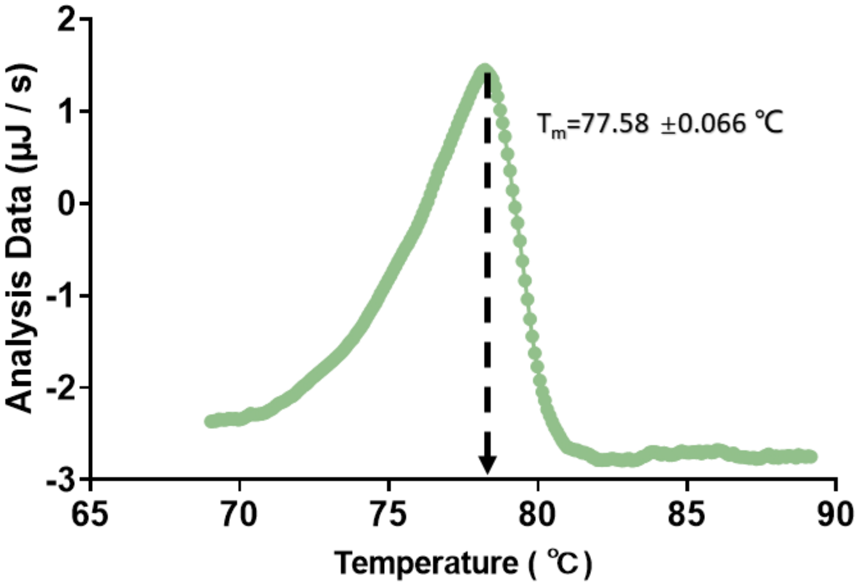

Supplementary Figure 7. The melting temperature (Tm) of *Kb*PETase was re-evaluated using nano DSC. The results indicated a Tm value of 77.58 ± 0.066 °C, which closely aligns with the value obtained through DSF, further validating the high thermal stability of *Kb*PETase.

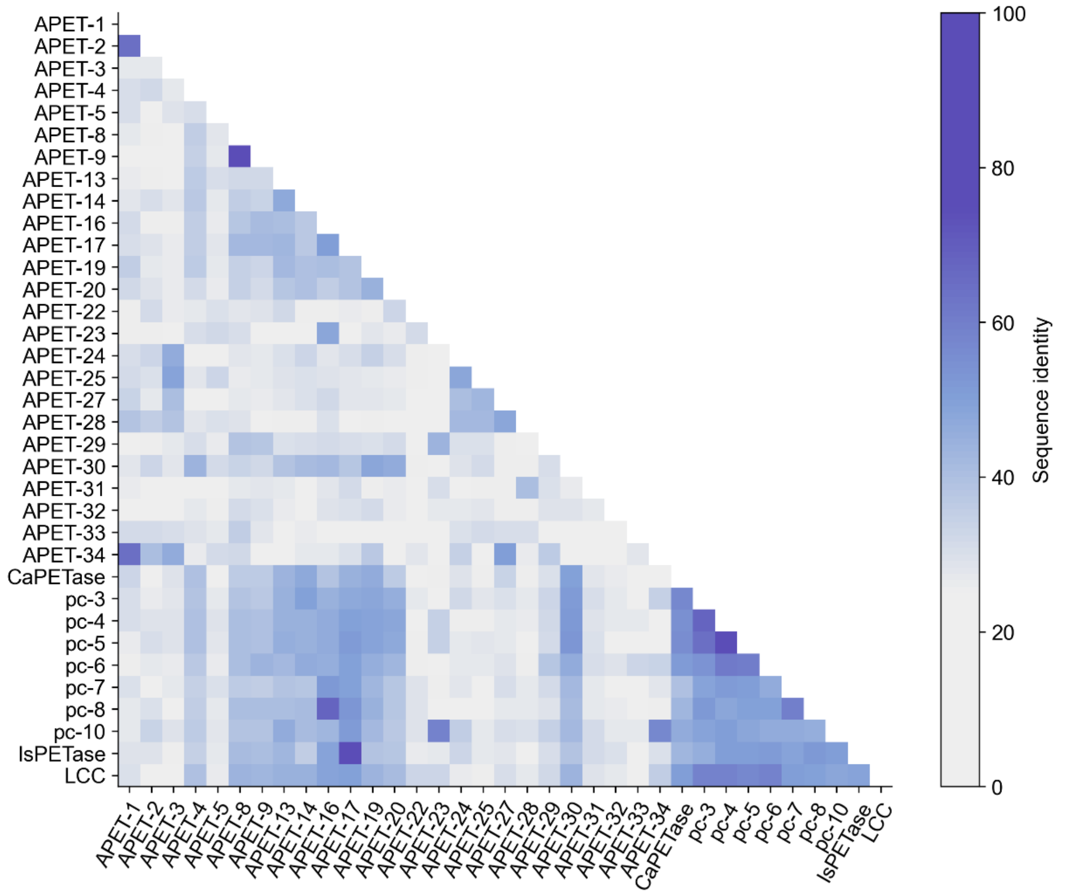

Supplementary Figure 8. The 34 candidate proteins identified as APET-1 to APET-34 were compared for sequence similarity with known PETases. Except for APET-17, which exhibited over 60% similarity, the sequence similarity of the other candidates to the template *Is*PETase ranged between 30% and 40%. Additionally, all candidates showed sequence similarities of 20% to 40% with LCC, indicating a relatively low level of sequence similarity overall. This approach enables the discovery of a substantial number of proteins with low sequence similarity, broadening the potential for enzyme engineering.

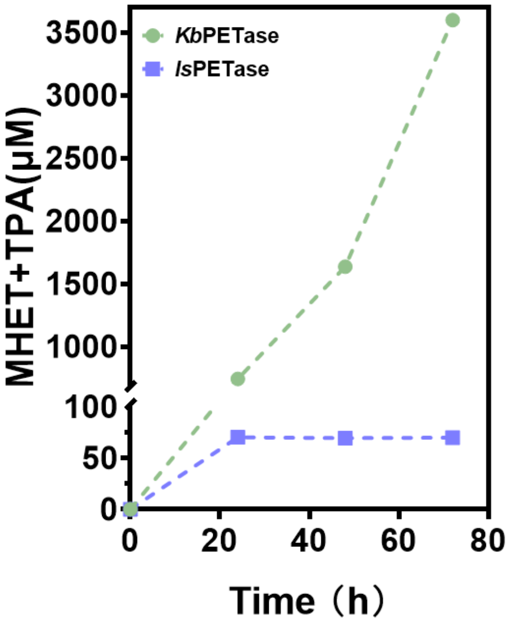

Supplementary Figure 9. The reaction time course profiles of *Is*PETase and *Kb*PETase. Comparison of the degradation activity of *Is*PETase and *Kb*PETase on PET films. The reaction was conducted in 50 mM Glycine-NaOH (pH 9.0) at their respective optimal temperatures (30°C for *Is*PETase and 50°C for *Kb*PETase) for 24, 48 and 72 h. Reactions were performed in triplicate; data are presented as mean values ± SD..

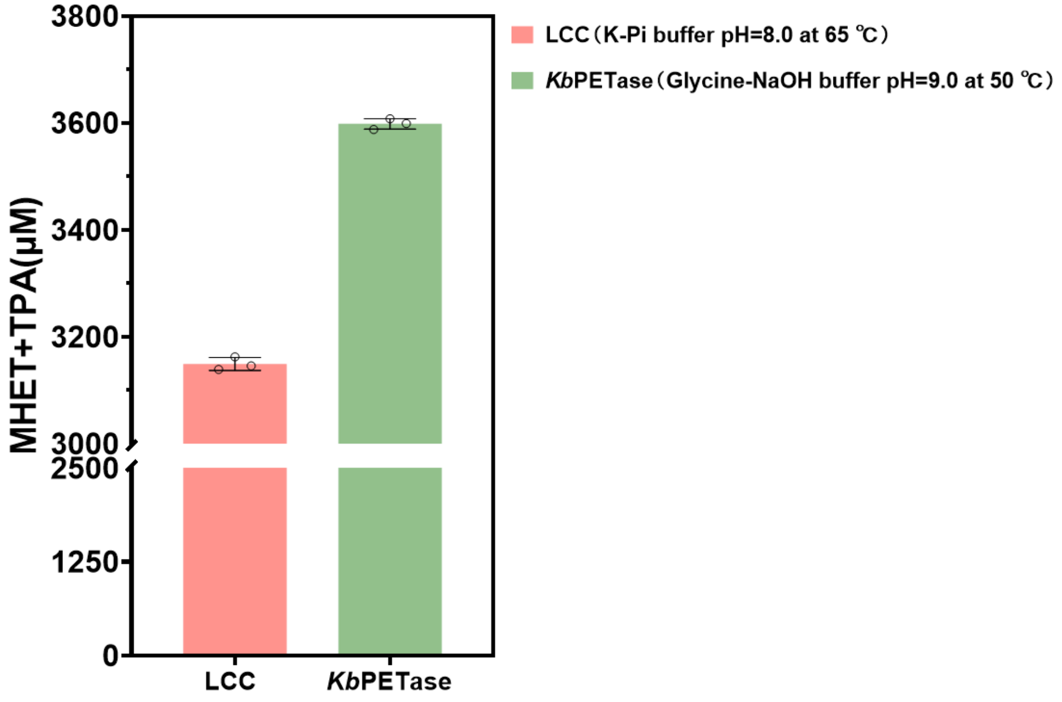

Supplementary Figure 10. Activity measurement of *Kb*PETase and LCC. PET film degradation activity of LCC compared to *Kb*PETase. The reactions of LCC and *Kb*PETase were respectively conducted in K-Pi buffer (pH 8.0) at 65°C and 50 mM Glycine-NaOH (pH 9.0) at 50 °C for 72 h. Reactions were performed in triplicate; data are presented as mean values ± SD.

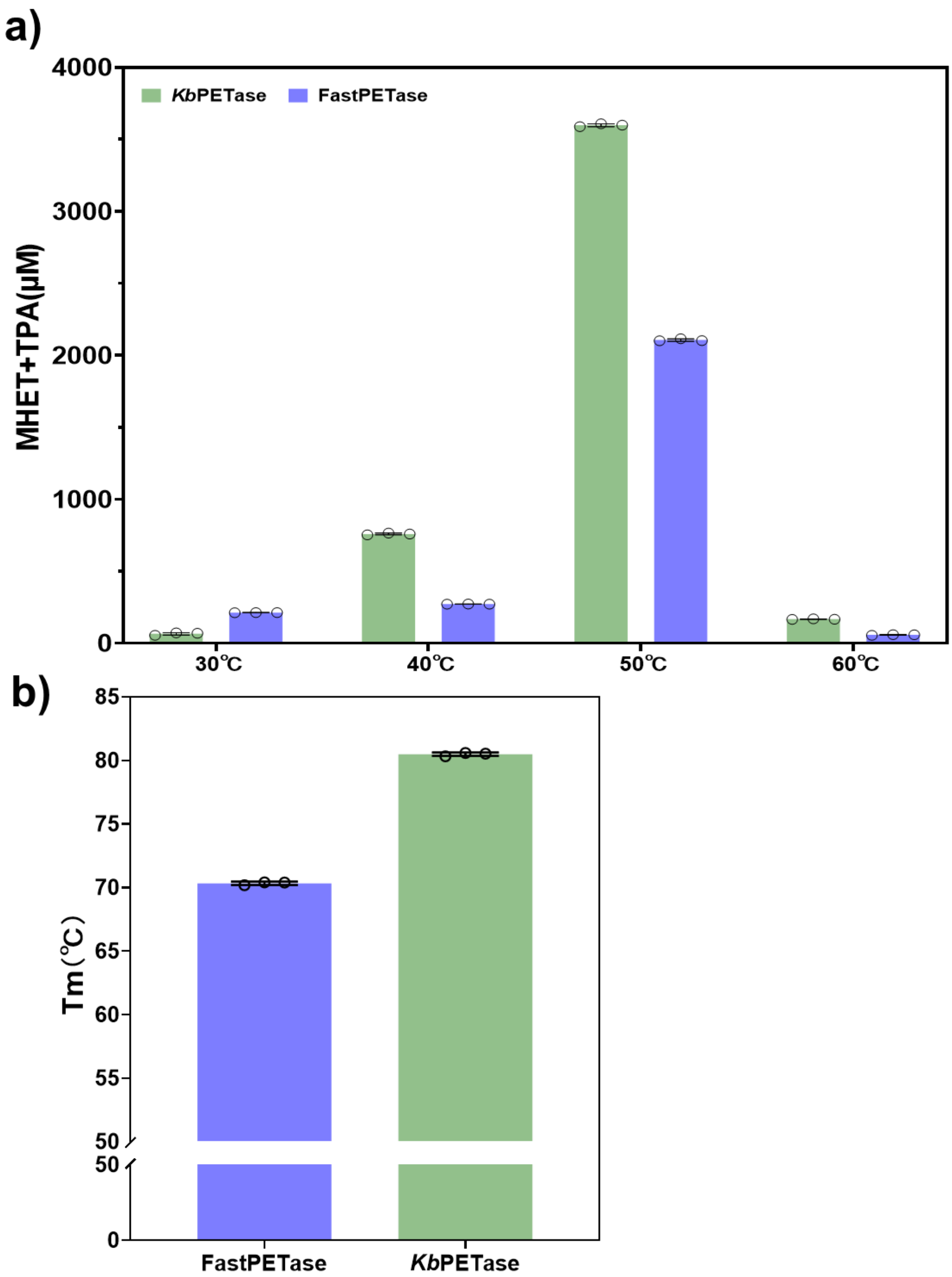

Supplementary Figure 11. Activity and thermostability measurements of *Kb*PETase and FastPETase**.** (a) PET film degradation activity of FastPETase compared to KbPETase. The reactions of FastPETase and *Kb*PETase were respectively conducted in K-Pi buffer (pH 8.0) and 50 mM Glycine-NaOH (pH 9.0) at 30 °C, 40 °C, 50 °C and 60 °C for 72 h.(b) Comparison of the Tm of *Kb*PETase with FastPETase using DSF. Reactions were performed in triplicate; data are presented as mean values ± SD.

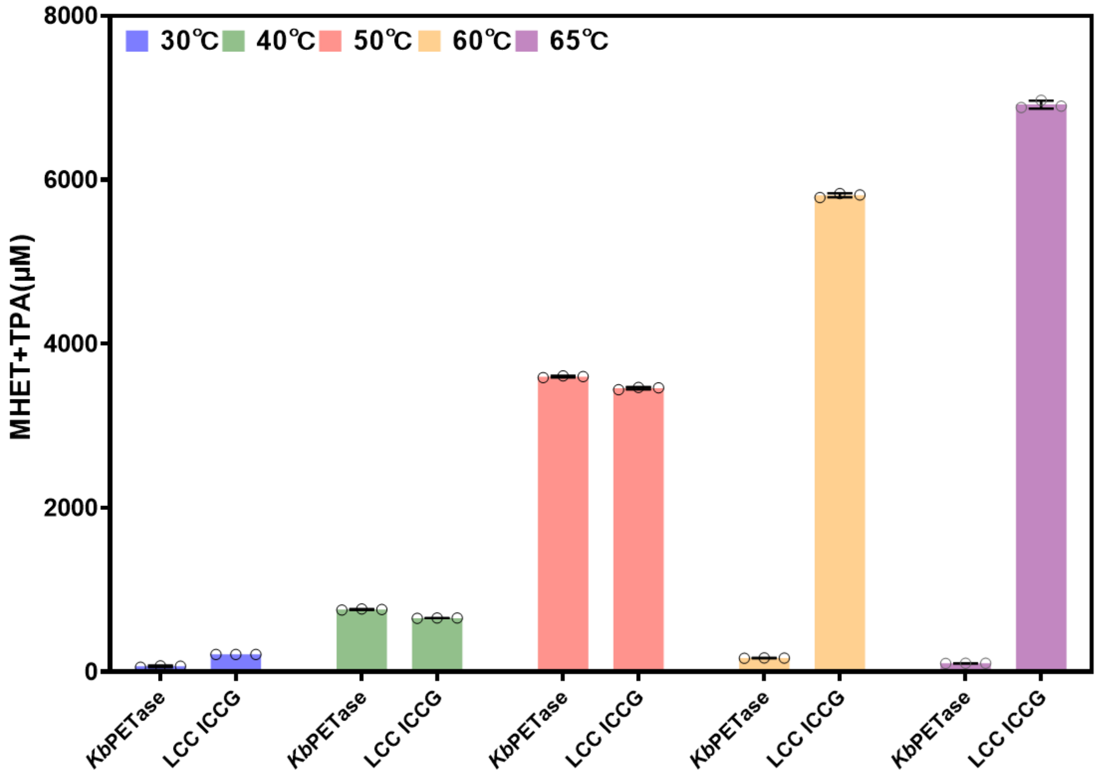

Supplementary Figure 12. Activity comparison of *Kb*PETase and ICCG. PET film degradation activity of ICCG and *Kb*PETase across a temperature range (30 ℃ to 65 ℃). Reactions were performed in K-Pi buffer (pH 8.0) for ICCG and 50 mM Glycine-NaOH (pH 9.0) for *Kb*PETase, incubated for 72 hours. Reactions were performed in triplicate; data are presented as mean values ± SD.

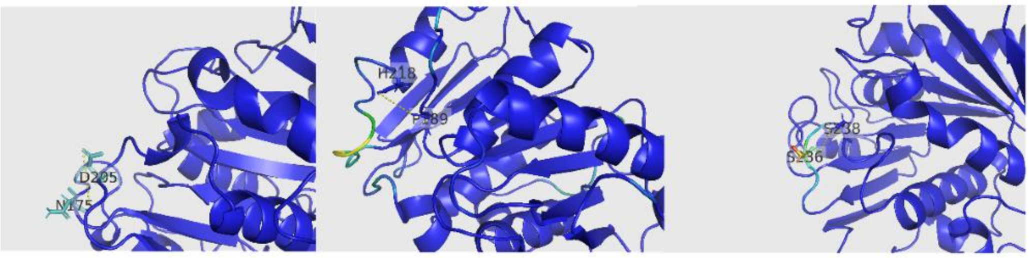

τ=5.02ns τ=4.35ns τ=3.36ns

Supplementary Figure 13. Lifetime analysis of critical catalytic pocket hydrogen bonds. *Kb*PETase (left) retains a stable N175-D205 hydrogen bond (τ = 5 ns), exceeding lifetimes τ = 4.35 ns in LCC (middle) and τ = 3.36ns *Is*PETase (right).

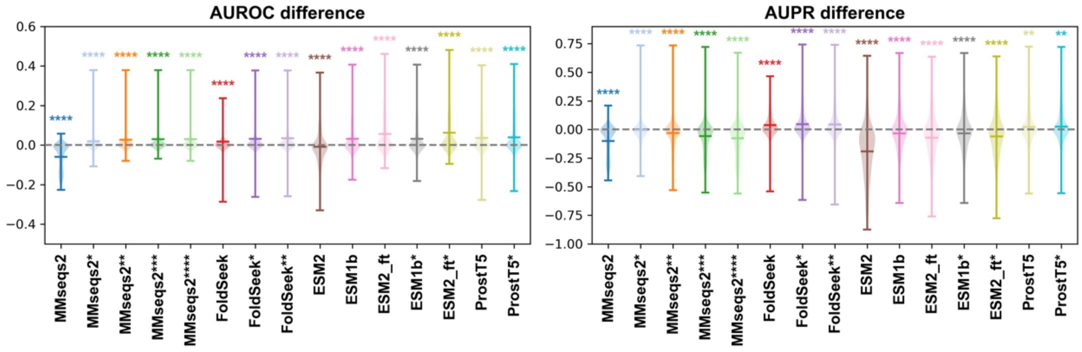

Supplementary Figure 14. Comparison of different models towards BLAST on AUROC and AUPR difference for protein discovery. Different methods are tested including MMseqs2 (various maxseq), FoldSeek (various maxseq), ESM2 (Euclidean distance or cosine similarity), ESM1b (Euclidean distance or cosine similarity), fine-tuned ESM2 (Euclidean distance or cosine similarity), and ProstT5 (Euclidean distance or cosine similarity). For language models, * are marked for cosine similarity. MMseqs2 including using different max sequence setting including 1000 (default), 2500 (*), 5000 (**), 10000 (**), 20000 (**). FoldSeek including 3 maxseq settings including 1000 (default), 2500 (*) and 5000 (**). Two sided Wilcoxon signed-rank test are employed to compute the significance of the difference if AUROC/AUPR between the target method and BLAST. The asterisks above the violin plot indicate the p-value for two-sided Wilcoxon sum rank test, ranging from 0.01~0.05 (*), 0.001~0.01 (**), 0.0001~0.001 (***), and <0.0001 (****).

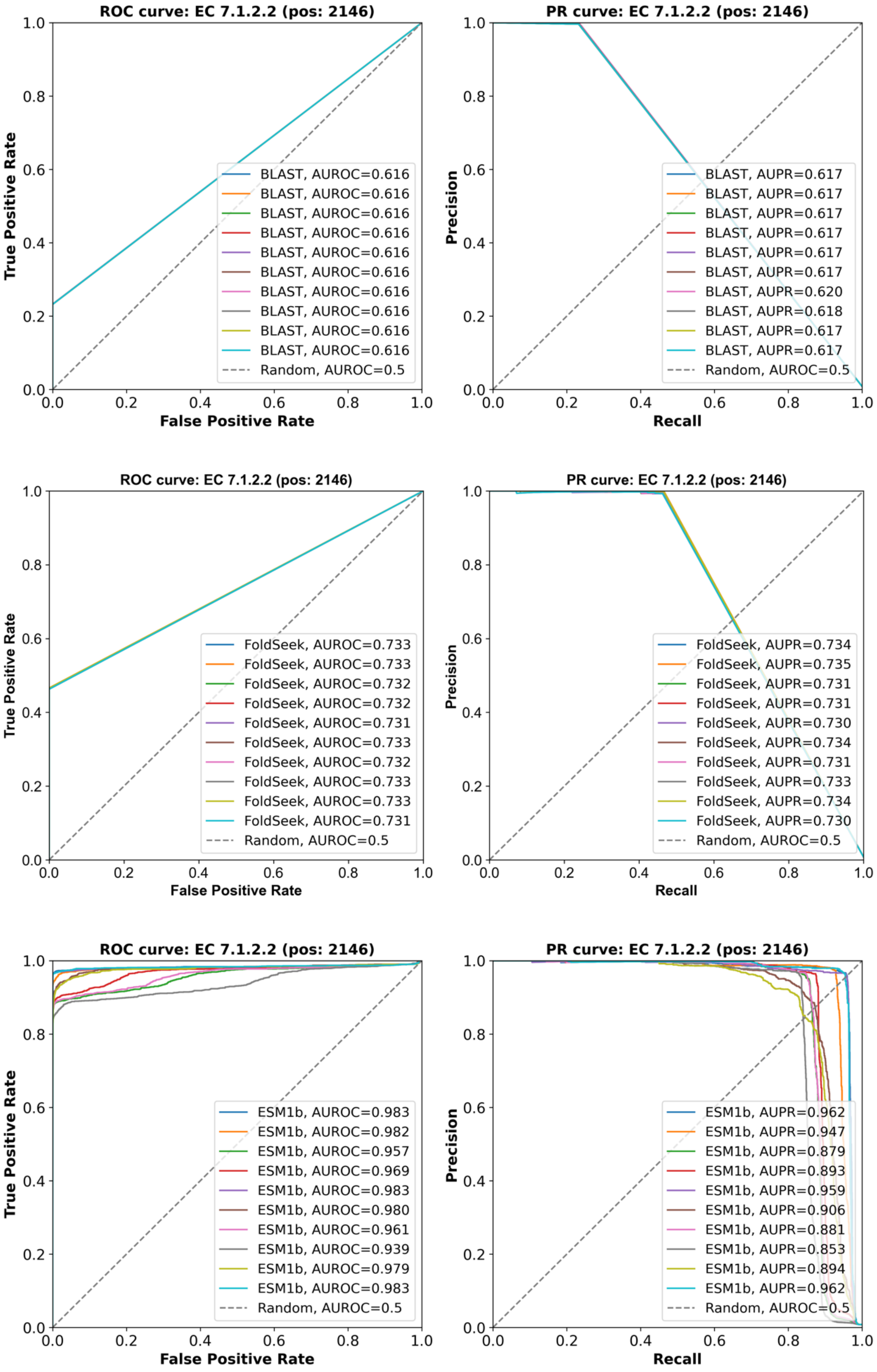

ROC curve: EC 7.1.2.2 (pos: 2146)
True Positive Rate
False Positive Rate
0.0
0.2
0.4
0.6
0.8
1.0
BLAST, AUROC=0.616
BLAST, AUROC=0.616
BLAST, AUROC=0.616
BLAST, AUROC=0.616
BLAST, AUROC=0.616
BLAST, AUROC=0.616
BLAST, AUROC=0.616
BLAST, AUROC=0.616
BLAST, AUROC=0.616
BLAST, AUROC=0.616
Random, AUROC=0.5
PR curve: EC 7.1.2.2 (pos: 2146)
Precision
Recall
BLAST, AUPR=0.617
BLAST, AUPR=0.617
BLAST, AUPR=0.617
BLAST, AUPR=0.617
BLAST, AUPR=0.617
BLAST, AUPR=0.617
BLAST, AUPR=0.620
BLAST, AUPR=0.618
BLAST, AUPR=0.617
BLAST, AUPR=0.617
Random, AUROC=0.5
ROC curve: EC 7.1.2.2 (pos: 2146)
True Positive Rate
False Positive Rate
FoldSeek, AUROC=0.733
FoldSeek, AUROC=0.733
FoldSeek, AUROC=0.732
FoldSeek, AUROC=0.732
FoldSeek, AUROC=0.731
FoldSeek, AUROC=0.733
FoldSeek, AUROC=0.732
FoldSeek, AUROC=0.733
FoldSeek, AUROC=0.733
FoldSeek, AUROC=0.731
Random, AUROC=0.5
PR curve: EC 7.1.2.2 (pos: 2146)
Precision
Recall
FoldSeek, AUPR=0.734
FoldSeek, AUPR=0.735
FoldSeek, AUPR=0.731
FoldSeek, AUPR=0.731
FoldSeek, AUPR=0.730
FoldSeek, AUPR=0.734
FoldSeek, AUPR=0.731
FoldSeek, AUPR=0.733
FoldSeek, AUPR=0.734
FoldSeek, AUPR=0.730
Random, AUROC=0.5
ROC curve: EC 7.1.2.2 (pos: 2146)
True Positive Rate
False Positive Rate
ESM1b, AUROC=0.983
ESM1b, AUROC=0.982
ESM1b, AUROC=0.957
ESM1b, AUROC=0.969
ESM1b, AUROC=0.983
ESM1b, AUROC=0.980
ESM1b, AUROC=0.961
ESM1b, AUROC=0.939
ESM1b, AUROC=0.979
ESM1b, AUROC=0.983
Random, AUROC=0.5
PR curve: EC 7.1.2.2 (pos: 2146)
Precision
Recall
ESM1b, AUPR=0.962
ESM1b, AUPR=0.947
ESM1b, AUPR=0.879
ESM1b, AUPR=0.893
ESM1b, AUPR=0.959
ESM1b, AUPR=0.906
ESM1b, AUPR=0.881
ESM1b, AUPR=0.853
ESM1b, AUPR=0.894
ESM1b, AUPR=0.962
Random, AUROC=0.5

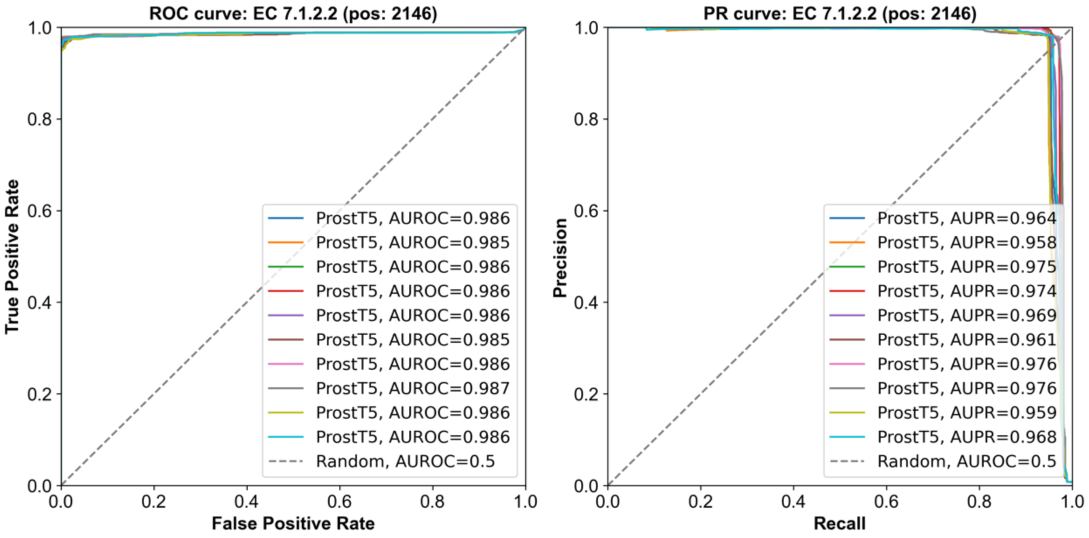

Supplementary Figure 15. The ROC curve and PR curve when evaluate the BLAST, FoldSeek, ESM-1b and ProstT5 ability to find enzymes with same EC number 7.1.2.2. Curves with 10 colors indicate the 10 randomly selected starting points.

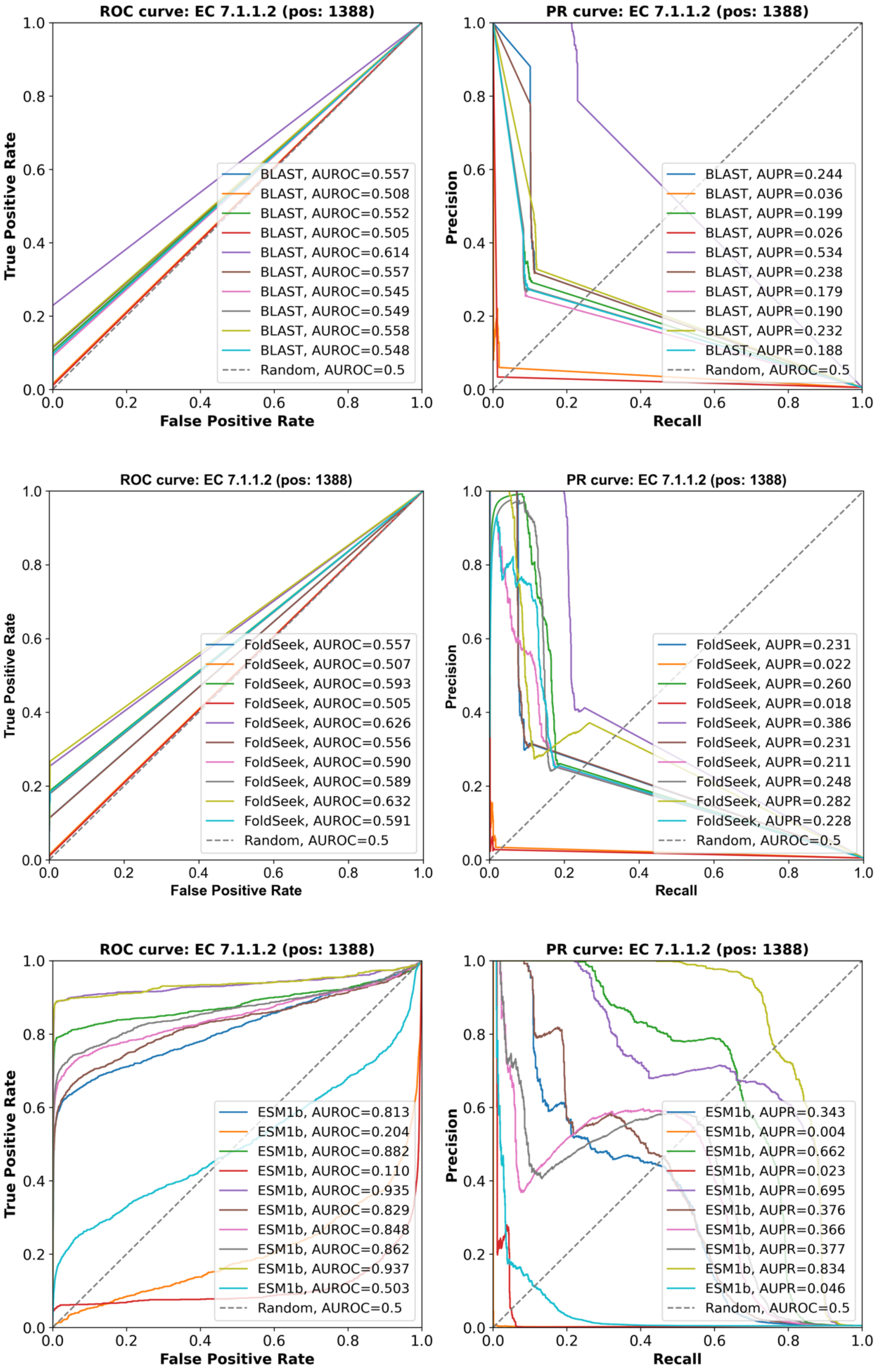
ROC curve: EC 7.1.1.2 (pos: 1388)
True Positive Rate
False Positive Rate
BLAST, AUROC=0.557
BLAST, AUROC=0.508
BLAST, AUROC=0.552
BLAST, AUROC=0.505
BLAST, AUROC=0.614
BLAST, AUROC=0.557
BLAST, AUROC=0.545
BLAST, AUROC=0.549
BLAST, AUROC=0.558
BLAST, AUROC=0.548
Random, AUROC=0.5
PR curve: EC 7.1.1.2 (pos: 1388)
Precision
Recall
BLAST, AUPR=0.244
BLAST, AUPR=0.036
BLAST, AUPR=0.199
BLAST, AUPR=0.026
BLAST, AUPR=0.534
BLAST, AUPR=0.238
BLAST, AUPR=0.179
BLAST, AUPR=0.190
BLAST, AUPR=0.232
BLAST, AUPR=0.188
Random, AUROC=0.5
ROC curve: EC 7.1.1.2 (pos: 1388)
True Positive Rate
False Positive Rate
FoldSeek, AUROC=0.557
FoldSeek, AUROC=0.507
FoldSeek, AUROC=0.593
FoldSeek, AUROC=0.505
FoldSeek, AUROC=0.626
FoldSeek, AUROC=0.556
FoldSeek, AUROC=0.590
FoldSeek, AUROC=0.589
FoldSeek, AUROC=0.632
FoldSeek, AUROC=0.591
Random, AUROC=0.5
PR curve: EC 7.1.1.2 (pos: 1388)
Precision
Recall
FoldSeek, AUPR=0.231
FoldSeek, AUPR=0.022
FoldSeek, AUPR=0.260
FoldSeek, AUPR=0.018
FoldSeek, AUPR=0.386
FoldSeek, AUPR=0.231
FoldSeek, AUPR=0.211
FoldSeek, AUPR=0.248
FoldSeek, AUPR=0.282
FoldSeek, AUPR=0.228
Random, AUROC=0.5
ROC curve: EC 7.1.1.2 (pos: 1388)
True Positive Rate
False Positive Rate
ESM1b, AUROC=0.813
ESM1b, AUROC=0.204
ESM1b, AUROC=0.882
ESM1b, AUROC=0.110
ESM1b, AUROC=0.935
ESM1b, AUROC=0.829
ESM1b, AUROC=0.848
ESM1b, AUROC=0.862
ESM1b, AUROC=0.937
ESM1b, AUROC=0.503
Random, AUROC=0.5
PR curve: EC 7.1.1.2 (pos: 1388)
Precision
Recall
ESM1b, AUPR=0.343
ESM1b, AUPR=0.004
ESM1b, AUPR=0.662
ESM1b, AUPR=0.023
ESM1b, AUPR=0.695
ESM1b, AUPR=0.376
ESM1b, AUPR=0.366
ESM1b, AUPR=0.377
ESM1b, AUPR=0.834
ESM1b, AUPR=0.046
Random, AUROC=0.5

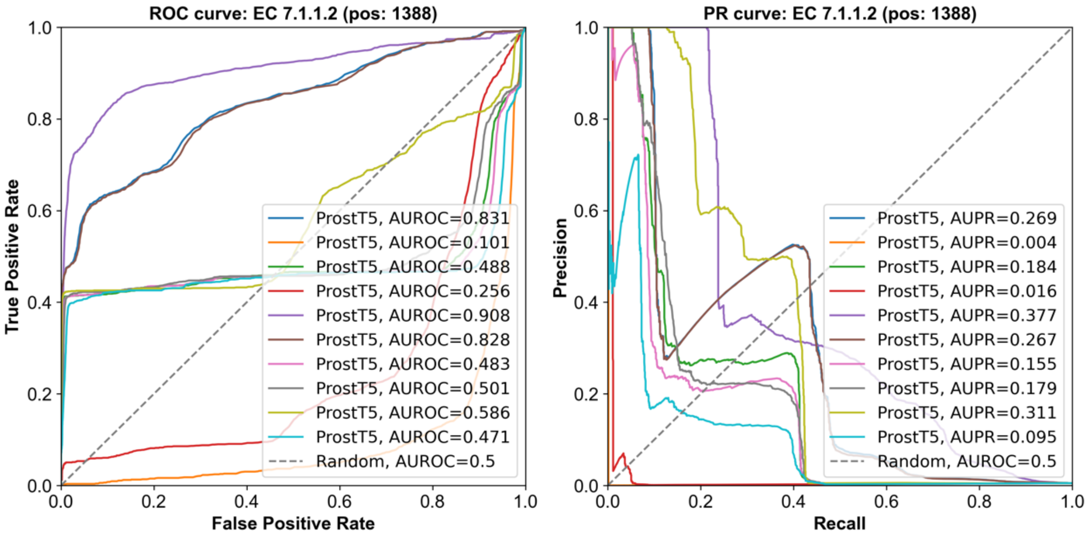

Supplementary Figure 16. The ROC curve and PR curve when evaluate the BLAST, FoldSeek, ESM-1b and ProstT5 ability to find enzymes with same EC number 7.1.1.2. Curves with 10 colors indicate the 10 randomly selected starting points.

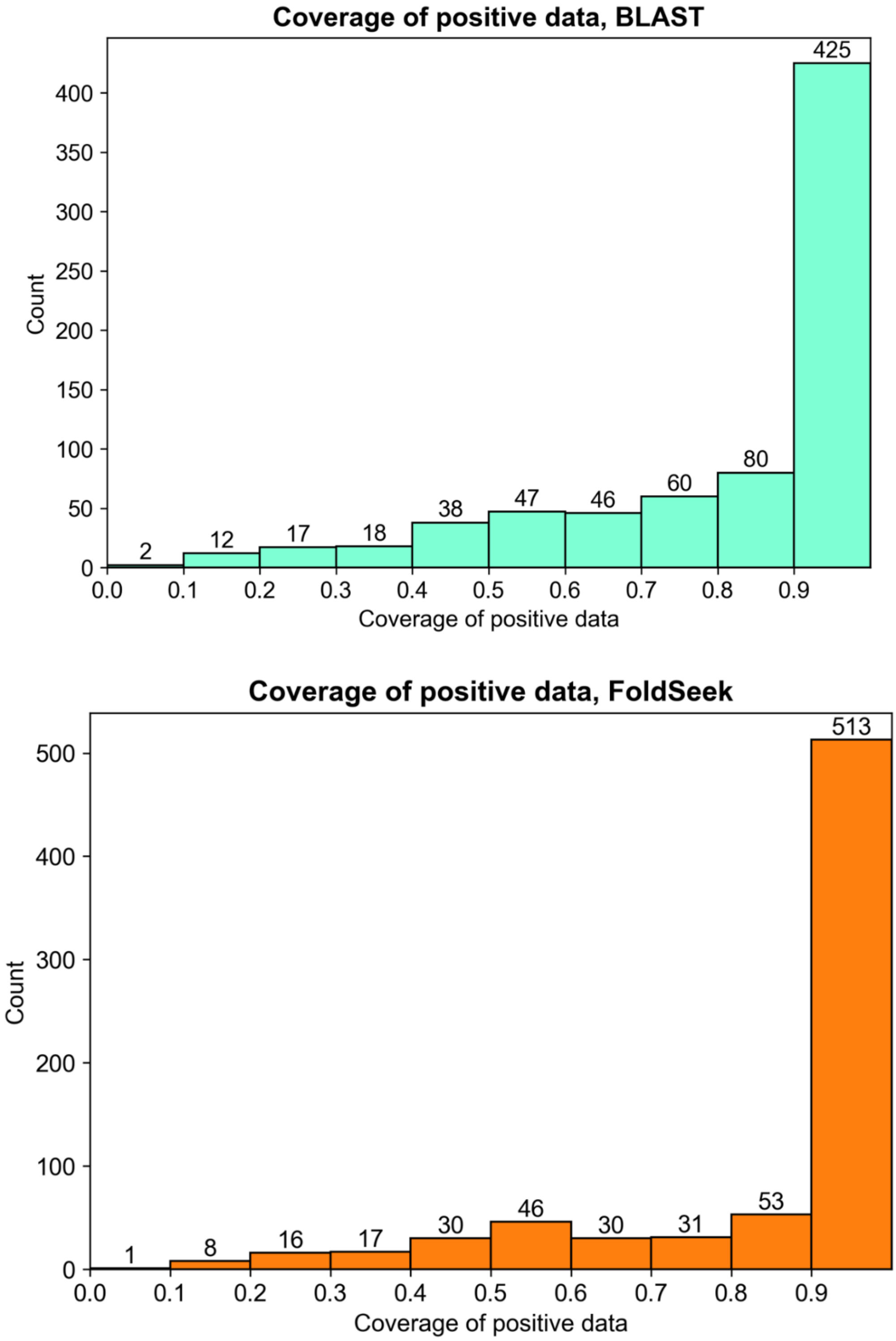

Supplementary Figure 17. Comparing the coverage of BLAST and FoldSeek founded positive candidates as a fraction of all positive candidates (true positive rate, recall). BLAST and FoldSeek both selected E-value > 10e-3 as cutoff. X-axis marked the recall range; y axis marked the count of EC number.

Supplementary table 1. Data collection and refinement statistics.

	*Kb*PETase
**Data collection**	
Wavelength (Å)	0.9792
Space group	P 1 21 1
Cell dimensions	
a, b, c (Å)	62.50, 104.80, 71.02
α, β, γ (▫)	90.0, 93.8, 90.0
Resolution (Å)	31.33 - 1.75 (1.81 - 1.75)
R-merge	0.06559 (0.5443)
$I/\sigma I$	13.42(3.54)
Completeness (%)	96.96 (94.87)
Redundancy	2.9 (3.0)
CC (1/2)	0.997 (0.649)
**Refinement**	
Resolution (Å)	31.33 - 1.75
No.reflections	258810 (26166)
R-work/R-free	0.1647 (0.2686)/ 0.1817 (0.3026)
No. atoms	
Protein residues	1004
Water	823
Mean B-factor ($Å^2$)	20.84
Protein	19.75
Water	30.69
RMS deviation bond lengths (Å)	0.014
RMS deviation bond angles (▫)	1.60
Ramachandran plot	
Favored regions (%)	97.59
Allowed regions (%)	2.41
Outliers regions (%)	0.00
Clashscore	3.44

Statistics for the highest-resolution shell are shown in parentheses.

References:

1 Almagro Armenteros, J. J. *et al.* SignalP 5.0 improves signal peptide predictions using deep neural networks. *Nature Biotechnology* **37**, 420-423, doi:10.1038/s41587-019-0036-z (2019).

2 Furukawa, M., Kawakami, N., Tomizawa, A. & Miyamoto, K. Efficient Degradation of Poly(ethylene terephthalate) with Thermobifida fusca Cutinase Exhibiting Improved Catalytic Activity Generated using Mutagenesis and Additive-based Approaches. *Scientific Reports* **9**, 16038, doi:10.1038/s41598-019-52379-z (2019).
