# Supplementary material for: Harnessing protein language model for structure-based discovery of highly efficient and robust PET hydrolases
Source: Nat Commun. 2025 Jul 5;16:6211. doi: 10.1038/s41467-025-61599-z (PMC12228687; doi:10.1038/s41467-025-61599-z)
Supplement: Supplementary file 2 — Description of Additional Supplementary Files [file 41467_2025_61599_MOESM2_ESM.pdf]

Description of Additional Supplementary Files

File Name: Supplementary Data 1.

Description: Nucleotide sequences of APET1-34.

File Name: Supplementary Data 2.

Description: Wild type sequences in selected clusters
